# Global and regional child deaths due to injuries: an assessment of the evidence

**DOI:** 10.7189/jogh.08.021104

**Published:** 2018-12

**Authors:** Davies Adeloye, Kirsty Bowman, Kit Yee Chan, Smruti Patel, Harry Campbell, Igor Rudan

**Affiliations:** Centre for Global Health Research, University of Edinburgh, Medical School, Edinburgh, Scotland, UK

## Abstract

**Background:**

Injuries result in substantial number of deaths among children globally. The burden across many settings is largely unknown. We estimated global and regional child deaths due to injuries from publicly available evidence.

**Methods:**

We searched for community-based studies and nationally representative data reporting on child injury deaths published after year 1990 from CINAHL, EMBASE, IndMed, LILACS, Global Health, MEDLINE, SCOPUS, and Web of Science. Specific and all-cause mortality due to injuries were extracted for three age groups (0-11 months, 1-4 years, and 0-4 years). We conducted random-effects meta-analysis on extracted crude estimates, and developed a meta-regression model to determine the number of deaths due to injuries among children aged 0-4 years globally and across the World Health Organization (WHO) regions.

**Results:**

Twenty-nine studies from 16 countries met the selection criteria. A total of 230 data-points on 15 causes of injury deaths were retrieved from all studies. Eighteen studies were rated as high quality, although heterogeneity was high (I^2^ = 99.7%, *P* < 0.001) reflecting variable data sources and study designs. For children aged 0-11 months, the pooled crude injury mortality rate was 29.6 (95% confidence interval (CI) = 21.1-38.1) per 100 000 child population, with asphyxiation being the leading cause of death (neonatal) at 189.1 (95% CI = 142.7-235.4) per 100 000 followed by suffocation (post-neonatal) at 18.7 (95% CI = 11.8-25.7) per 100 000. Among children aged 1-4 years, the pooled crude injury mortality rate was 32.7 (95% CI = 27.3-38.1) per 100 000, with traffic injuries and drowning the leading causes of deaths at 10.8 (95% CI = 8.9-12.8) and 8.8 (95% CI = 7.5-10.2) per 100 000, respectively. Among children under five years, the pooled injury mortality rate was 37.7 (95% CI = 32.7-42.7) per 100 000, with traffic injuries and drowning also the leading causes of deaths at 10.3 (95% CI = 8.8-11.8) and 8.9 (95% CI = 7.8-9.9) per 100 000 respectively. When crude mortality changes over age, WHO regions, and study period were accounted for in our model, we estimated that in 2015 there were 522 167 (95% CI = 395 823-648 630) deaths among children aged 0-4 years, with South East Asia (SEARO) recording the highest number of deaths at 195 084 (95% CI = 159476-230502), closely followed by the Africa region (AFRO) with 176523 (95% CI = 115 040-237 831) deaths. Globally, traffic injuries and drowning were the leading causes of under-five injury fatalities in 2015 with 142 661 (22.0/100 000) and 123 270 (19.0/100 000) child deaths, respectively. The exception being burns in AFRO with 57 784 deaths (38.6/100 000).

**Conclusions:**

Varying study designs, case definitions, and particularly limited country representation from Africa and South-East Asia (where we reported higher estimates), imply a need for more studies for better population representative estimates. This study may have however provided improved understanding on child injury death profiles needed to guide further research, policy reforms and relevant strategies globally.

Several children die each year from injuries, assault or violence, with many suffering from consequences of non-fatal injuries [1]. In 2015, there were 7.3 million deaths among children and adolescents [2], with low- and middle-income countries (LMICs) accounting for 80%-95% of all fatalities in this age group [1,3]. Although, the global burden of disease (GBD) collaborators reported that global child mortality decreased by about 50% between 1990 and 2015 [2], these were mainly due to a reduction in infectious causes. Injuries are known to contribute less to global childhood mortality in comparison to known leading causes like pneumonia, diarrhea, preterm births, neonatal infections, or malnutrition. However, a steady decline in infectious causes with limited knowledge on the burden of child injuries across world regions implies injuries may constitute a hidden burden [4,5]. According to the World Health Organization (WHO), between 2000 and 2010, percentage distribution of mortality from injuries among children under five years increased across all income groups, suggesting a need to address this more keenly globally [1].

One basic challenge in child injuries’ research and surveillance relates to the application of appropriate case definitions [6,7]. Largely, case definitions, coding, and overall design of child injury studies vary across world regions [3,6]. Injuries are mainly classified by intent, *ie.* those that were not predetermined (unintentional), or those that were planned (intentional) [8]. The International Classification of Disease 10 (ICD-10) [9], and the International Classification of External Causes of Injuries (ICECI) have provided standard codes and definitions to guide researchers in describing and identifying injury types [10]. In several surveys however, researchers appear to have been limited in appropriately classifying and reporting injuries, with this resulting misrepresentation of cases and/or inconclusive outcomes [11-13].‬‬‬‬‬‬‬‬‬‬‬‬‬‬‬‬‬‬‬‬‬‬‬‬‬‬‬‬‬‬‬‬‬‬‬‬‬‬‬‬‬‬‬‬‬‬‬‬‬‬‬‬‬‬‬‬‬‬‬‬‬‬‬‬‬‬‬‬‬‬‬‬‬‬‬‬‬‬‬‬

Moreover, despite various sources available for conducting national and subnational injuries surveillance, including death certificates, autopsy reports, hospital reports, insurance records, and police records [12,14], data on unintentional injuries are still collated from very limited sources – mainly manually from hospital records across many LMICs [15]. Due to unclear reporting procedures, limited technical capacity for electronic collation, and failure to account for deaths outside hospital settings, data are almost always incomplete and estimates are largely not representative [8,16]. With prevailing sparse data across many countries, it remains difficult to convince policy makers and relevant stakeholders on the magnitude of child injuries [1,3]. There are several doubts and persisting uncertainties around the global burden of child deaths from injuries, nonetheless newer studies are emerging in some settings [17]. Thus, retrieving the available data across world regions through a comprehensive and systematic search is imperative to guide better understanding of the burden, and possibly aid relevant policy decisions, actions and improved research in settings with limited data. We sought to provide estimates of the global and regional mortality and absolute number of deaths from injuries among children under five years from publicly available evidence.

## METHODS

### Search strategy

A systematic literature search of CINAHL, EMBASE, IndMed, LILACS, Global Health, MEDLINE, SCOPUS and Web of Science was conducted for studies reporting mortalities from injuries among children under the age of five years across world regions. Search dates were set from January 1990 to August 2018. A further search of OpenGrey, BIOSIS and relevant children international organizations was conducted for grey literature and conference abstracts. Reference lists of initially identified studies were hand-searched for more studies. Full search strategy and search terms are presented in Table S1 in **Online Supplementary Document(Online Supplementary Document)**.

### Selection criteria

Articles were selected if they were i) community-based studies or nationally representative data on mortality from child injuries, ii) conducted among children under the age of five years (or where this could possibly be extracted if this age group was included in a broader age range), and iii) published from the year 1990 onwards. For the definition of injury, we considered articles that described injuries as physical hurt or damage to the body resulting from intentional of unintentional causes including (but not limited to) traffic collisions, drowning, poisoning, suffocation, falls, burns, violence, assaults or homicide [9]. We excluded studies if they were i) conducted among children above five years only, ii) conducted on specific groups of children with underlying conditions that could make children more prone to injuries, iii) reporting on non-fatal outcomes from injuries, iv) published before the year 1990, and/or v) reviews, commentaries, editorials or viewpoints.

### Data extraction

Two reviewers (DA and KB) independently screened studies against the selection criteria and extracted data from all selected studies. Any disagreements over article inclusion, exclusion or data extraction between the two reviewers were resolved through a final assessment by a third reviewer (IR). As we already employed a two-stage search and extraction process based on a combination of independent review and reassessment, we did not calculate Kappa statistics to determine agreement between the reviewers. We extracted data and relevant information systematically from each study. This included study location, period, design, location, WHO region, income category, coding of injury types, and corresponding deaths, population denominators, and injury mortality rates, respectively for ages 0-11 months, 1-4 years and 0-4 years. All extracted data were sorted in Excel Worksheet 2013 (Microsoft Inc, Redmond, WA, USA).

### Quality criteria

We assessed quality of studies using a modified approach of the Centre for Reviews and Dissemination guidance for undertaking reviews in health care [18]. The assessment was based on four criteria: study design (causes of injuries reported with appropriate coding), sampling (representative of national population), statistical analysis (appropriate for child injury mortality estimation), and study limitation (description of potential sources of bias). See **Table 1** for grading details.

**Table 1:** Quality assessment criteria

Criteria	Assessment	Score
Study design (At least 10 injury types reported and based on standard definitions or ICD coding?)	Yes	1
	No	0
Sampling (was it representative of target sub-national population or national population?)	Nationally representative	2
	Sub-nationally representative	1
	No	0
Statistical analysis (was it clear and appropriate for outcome measure?)	Yes	1
	Ambiguous	0
Study limitations (were potential sources of bias described)	Yes	1
	No	0
Final assessment: High (4-5), Moderate (2-3), and low (0-1)		

### Data analysis

For the three age groups, data were primarily sorted according to WHO regions: Africa (AFRO), Eastern Mediterranean (EMRO), Europe (EURO), Americas (PAHO), South East Asia (SEARO) and Western Pacific (WPRO). For the mechanism of injuries, data were sorted according to estimates for overall injuries, and individual injury types from each study. Mortality rates were estimated as number of child injury deaths per 100 000 child population. Standard errors were estimated from the crude mortality rates and child population assuming a Poisson distribution. Using a random effects meta-analysis (Der-Simonian and Laird method) [19], crude meta-estimates and confidence intervals were pooled from individual crude mortality rates and reported by injury-type and WHO regions. I-squared (I^2^) statistics and subgroup analysis were conducted to assess heterogeneity between studies. From reported crude mortality rates, we developed a meta-regression model to account for the absolute number of injury deaths among children under five years. In this model, we created six dummy variables accounting for each of the six WHO regions mainly from data that reported estimates for all injuries. We ran this regression model separately for each of the three age groups, while accounting for the study period and WHO regions. We tested the model using different WHO dummy region variables and chose the one that was most predictive (*ie.* AFRO, in which the proportion of variance (adjusted R^2^) of child injury mortality explained by WHO regions and study period was the greatest) as the control against which other variables (regions) were compared. Using a standardized ratio from the pooled crude mortality rates for seven causes of injuries that returned highest number of data-points (*ie*. traffic injuries, drowning, falls, poisoning, suffocation, burns, and assaults, with the remaining injury types grouped as “others”), we determined the number of deaths for each cause of injury by WHO regions. We summed these regional estimates to determine the absolute number of deaths for all injuries and specific causes of injuries among children aged 0-4 years worldwide. All statistical analyses were conducted on Stata 14 (Stata Corp LP, College Station, TX, USA).

## RESULTS

### Search results

**Figure 1:**
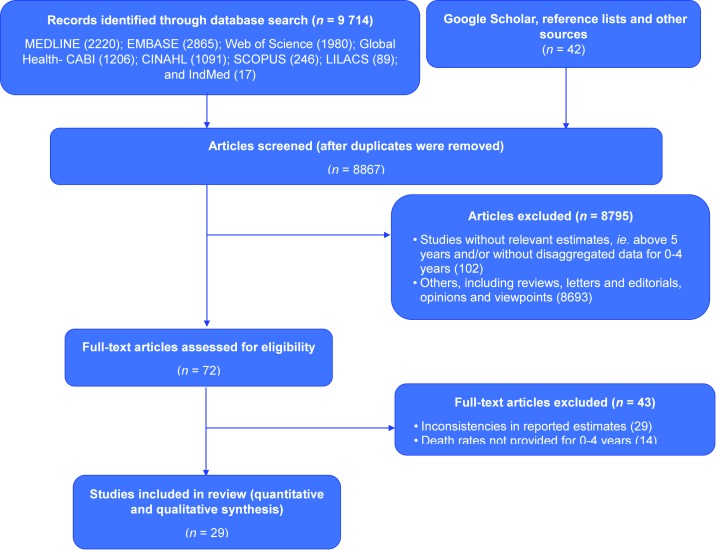
Flowchart of search strategy and study selection.

The literature search returned 9714 records from the electronic databases and 42 records from other sources. Following the removal of duplicates, screening of titles and abstracts, and the application of the selection criteria, 29 studies were selected. The process of selection of studies is described in **Figure 1**.

### Main study characteristics

**Figure 2:**
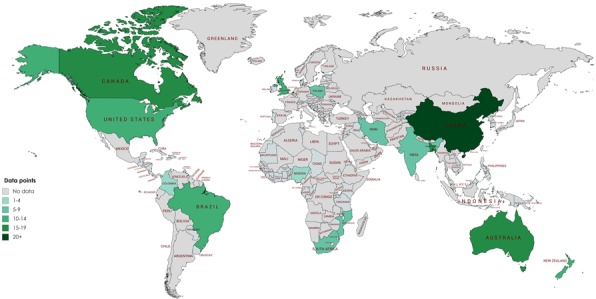
Sources of data on child injury deaths by country.

The 29 studies were selected across 16 countries (**Figure 2**), with a total of 230 data points on different injury types among children aged 0-4 years (**Table 2**). Most data were from WPRO with 105 data points and China accounting for 73.3% of this (**Figure 2**). PAHO had 54 data-points, SEARO 24, AFRO 20, EURO 19 and EMRO 8. Traffic injuries and drowning had most data points (n = 31 each), followed by falls (n = 27), poisoning (n = 22), suffocation (n = 21), burns (n = 15) and assault (n = 15) (**Table 2**). Most studies were population-based injury surveillance, with study period ranging from 1990 to 2017. Heterogeneity was high across studies (I^2^ = 99.7%, *P* < 0.001), mainly due to varying case definitions, coding and overall study designs. Eighteen studies were rated as high quality with the remaining 11 rated as moderate quality (**Table 3** **and** Table S2 in **Online Supplementary Document(Online Supplementary Document)**). All low-quality studies were not included in the review (see Table S3 in **Online Supplementary Document(Online Supplementary Document)** for details of low-quality studies excluded).

**Table 2:** Data distribution by injury type and WHO region

Injury type	AFRO	EMRO	EURO	PAHO	SEARO	WPRO	Total
**All Injuries** (V01-X59.9, Y85-Y86)	4	1	2	5	1	14	27
**Traffic Injuries** (V01-V99)	3	1	2	7	3	15	31
**Drowning (**W65-W74.9, V90-V90.0, and V92-V92.9)	2	1	2	6	3	17	31
**Burns** (X00-X19.9)	3	1	1	6	2	2	15
**Suffocation** (W75-W84)	1	0	1	4	2	13	21
**Falls** (W00-W19.9)	3	1	2	4	3	14	27
**Poisoning** (X40-X49.9)	1	1	2	2	1	15	22
**Firearm (**W32-W34.9)	0	0	1	2	0	2	5
**Cutting/Piercing** (X71-X83)	0	0	1	2	1	2	6
**Venomous Animals/Plants** (X20-X29)	0	1	0	0	3	0	4
**Electrocution** (W85-W99)	1	0	0	1	1	0	3
**Asphyxiation (**T71.1)	0	0	0	1	1	1	3
**Medical** Procedures (Y84.8)	1	1	1	0	0	1	4
**Unspecified unintentional injuries**	0	0	2	3	2	2	9
**Assault/Homicide** (X85-Y09)	1	0	1	7	1	5	15
**Intent Unknown**	0	0	1	4	0	2	7
**Total**	**20**	**8**	**19**	**54**	**24**	**105**	**230**

AFRO – African region, EMRO – Eastern Mediterranean region, EURO – European region, PAHO – Pan-American Health Organization or American region, SEARO – South East Asia region, WPRO – Western Pacific region

**Table 3:** Characteristics of selected studies

First author	Study period	Country	Income category	Study design	Quality grading
**AFRO**					
**Nizamo** [20]	2000	Mozambique	Low	Population based. Registered deaths	Moderate
**Abdur-Rahman** [21]	2015	Nigeria	Lower middle	Cross-sectional Verbal autopsy results	Moderate
**Norman Pacella** [22]	2000	South Africa	Upper middle	Population based. Death certificates	Moderate
**Sacarlal** [23]	2006	Mozambique	Low	Cross-sectional survey. Verbal autopsy results	Moderate
**EMRO**					
**Naghavi** [24]	2005	Iran	Upper middle	Population based. Death registration data	Moderate
**EURO**					
**DiGuiseppi** [25]	1992	England & Wales	High	Population based. WHO European detailed mortality database	High
**Grajda** [26]	1999-2012	Poland	High	Population based. WHO European detailed mortality database	High
**PAHO**					
**Celis** [27]	1997	Mexico	Upper middle	Population based. Death certificates	High
**D'Agostini** [28]	1996-2002	Brazil	Upper middle	Ecological model. Mortality data from mortality information system	High
**Espitia-Hardeman** [29]	2007	Colombia	Upper middle	Population based. Injury surveillance system	High
**Fingerhut** [30]	1993	USA	High	Population based. Injury surveillance system	High
**Gawryszewski** [31]	2003	Brazil	Upper middle	Population based. Mortality data from mortality information system	High
**Aldana** [32]	2009	Colombia	Upper middle	Population based. Death certificates	High
**Amram** [33]	2009	Canada	High	Population based. Injury surveillance system	High
**Clemens** [34]	2012	Canada	High	Population based. Injury surveillance system	High
**SEARO**					
**Rahman** [35]	2005	Bangladesh	Lower middle	Population based. Verbal autopsy results	High
**Jagnoor** [36]	2003	India	Lower middle	Cross-sectional. Verbal autopsy results	High
**Alonge** [37]	2017	Bangladesh	Lower middle	Population based. Injury surveillance system	High
**WPRO**					
**Huo** [38]	2004-2008	China	Upper middle	Population based. Annual reports of mortality data for maternal and child health	High
**Langley** [39]	1993	New Zealand	High	Population based. Injury surveillance system	High
**Lili** [40]	2009-2014	China	Upper middle	Population based. Disease surveillance information systems.	High
**Scott** [41]	1994	Australia	High	Population based. Child deaths registry	High
**Wallis** [42]	2008	Australia	High	Population based. Child deaths registry	High
**Wang** [43]	2000-2008	China	Upper middle	Population based. Child deaths obtained from Shenzhen Women and Child Health Surveillance System for 2004-2008	Moderate
**Yang** [44]	2001	China	Upper middle	Population based. Disease surveillance information systems.	Moderate
**Zhang** [45]	1997-2012	China	Upper middle	Population based. Child deaths registry	Moderate
**Zhang** [46]	2004-2010	China	Upper middle	Population based. Child deaths registry	Moderate
**Wang** [47]	2004-2008	China	Upper middle	Population based. Child deaths registry	Moderate
**Hayman** [48]	2002-2009	New Zealand	High	Population based. Disease surveillance information systems.	Moderate

AFRO – African region, EMRO – Eastern Mediterranean region, EURO – European region, PAHO – Pan-American Health Organization or American region, SEARO – South East Asia region, WPRO – Western Pacific region

### Pooled crude child injury mortality meta-estimates

**Figure 3:**
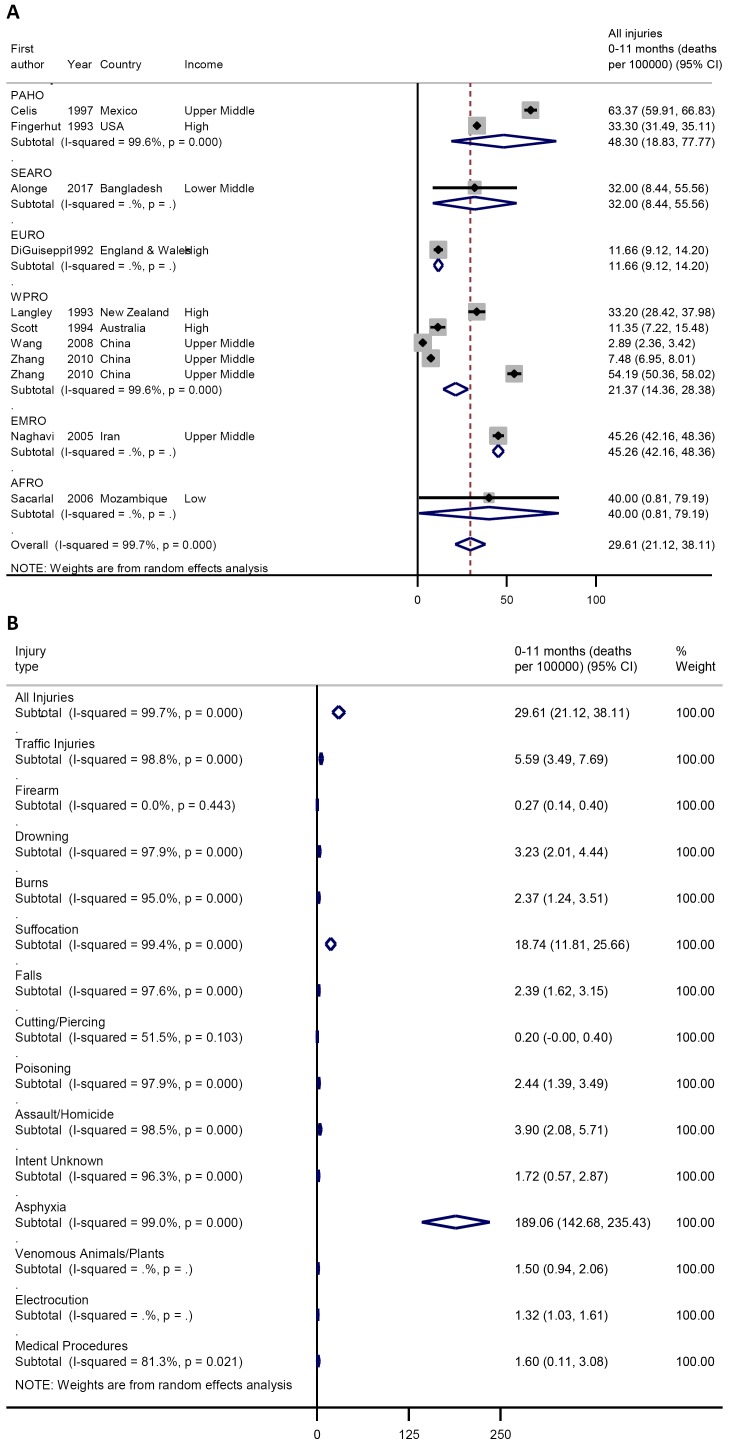
**A.** Pooled crude child injury mortality rate by WHO region, 0-11 months. **B.** Pooled crude child injury mortality rate by type of injury, 0-11 months.

For children aged 0-11 months, the pooled crude injury mortality rate was 29.6 (21.1-38.1) per 100 000 child population (**Figure 3****,** plate A), with asphyxiation being the leading cause of death (in the neonatal period) at 189.1 (95% confidence interval (CI) = 142.7-235.4) per 100 000 followed by suffocation (in the post-neonatal period) at 18.7 (95% CI = 11.8-25.7) per 100 000. Other notable causes of injury deaths in this age group include traffic injuries 5.6 (95% CI = 3.5-7.7) per 100 000, assault 3.6 (95% CI = 2.1-5.7) per 100 000, drowning 3.2 (95% CI = 2.0-4.4) per 100 000, and burns 2.4 (95% CI = 1.4-3.5) per 100 000 (**Figure 3****,** plate B).

**Figure 4:**
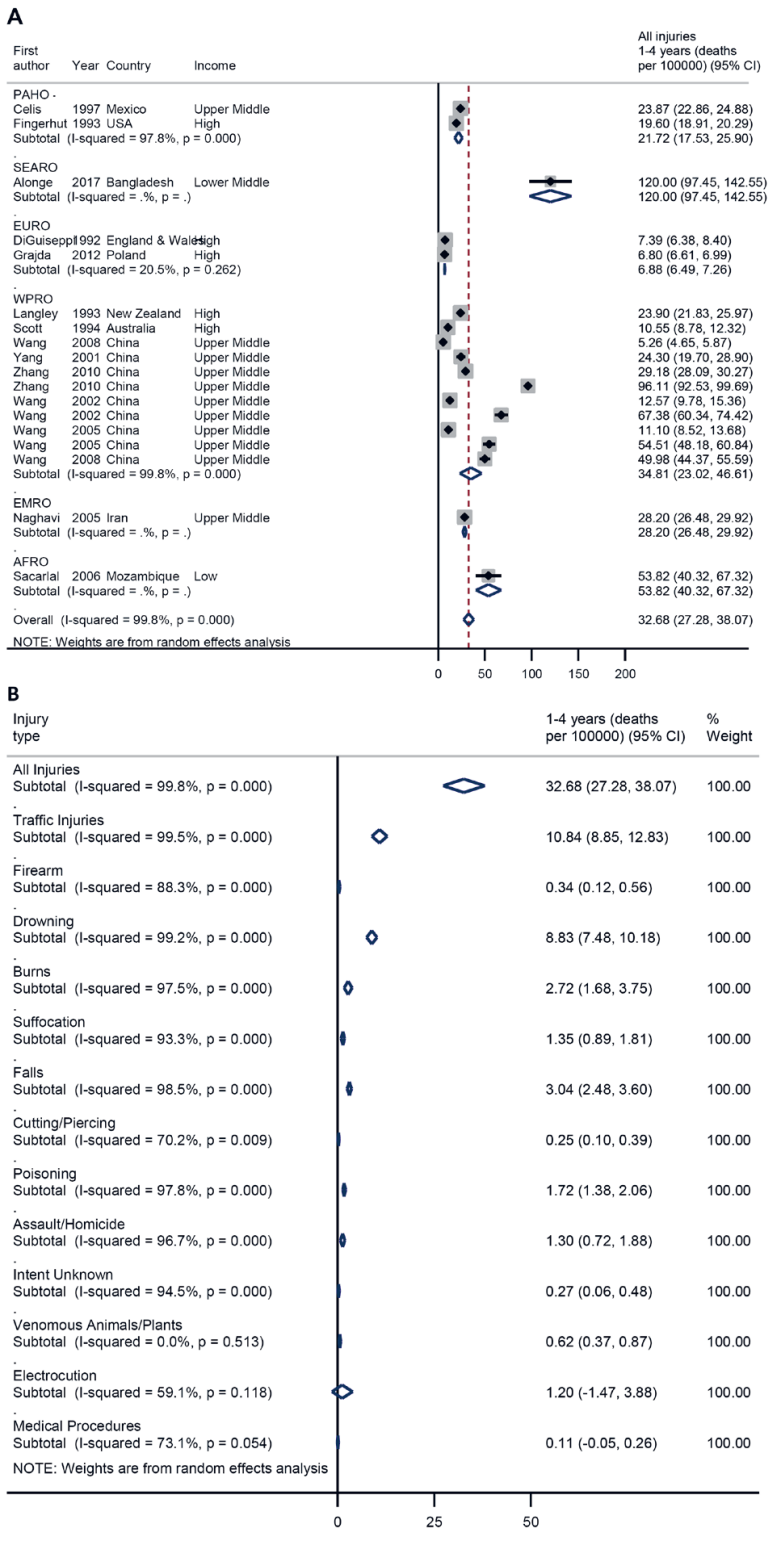
**A.** Pooled crude child injury mortality rate by WHO region, 1-4 years. **B.** Pooled crude child injury mortality rate by type of injury, 1-4 years.

Among children aged 1-4 years, the pooled crude injury mortality rate was 32.7 (27.3-38.1) per 100 000 (**Figure 4**, plate A). Traffic injuries and drowning the leading causes of deaths at 10.8 (95% CI = 8.9-12.8) and 8.8 (95% CI = 7.5-10.2) per 100 000, respectively. Other causes injury deaths include falls 3.0 (95% CI = 2.5-3.6), burns 2.7 (95% CI = 1.7-3.8) per 100 000, poisoning 1.7 (95% CI = 1.4-2.1) per 100 000 (**Figure 4**, plate B).

**Figure 5:**
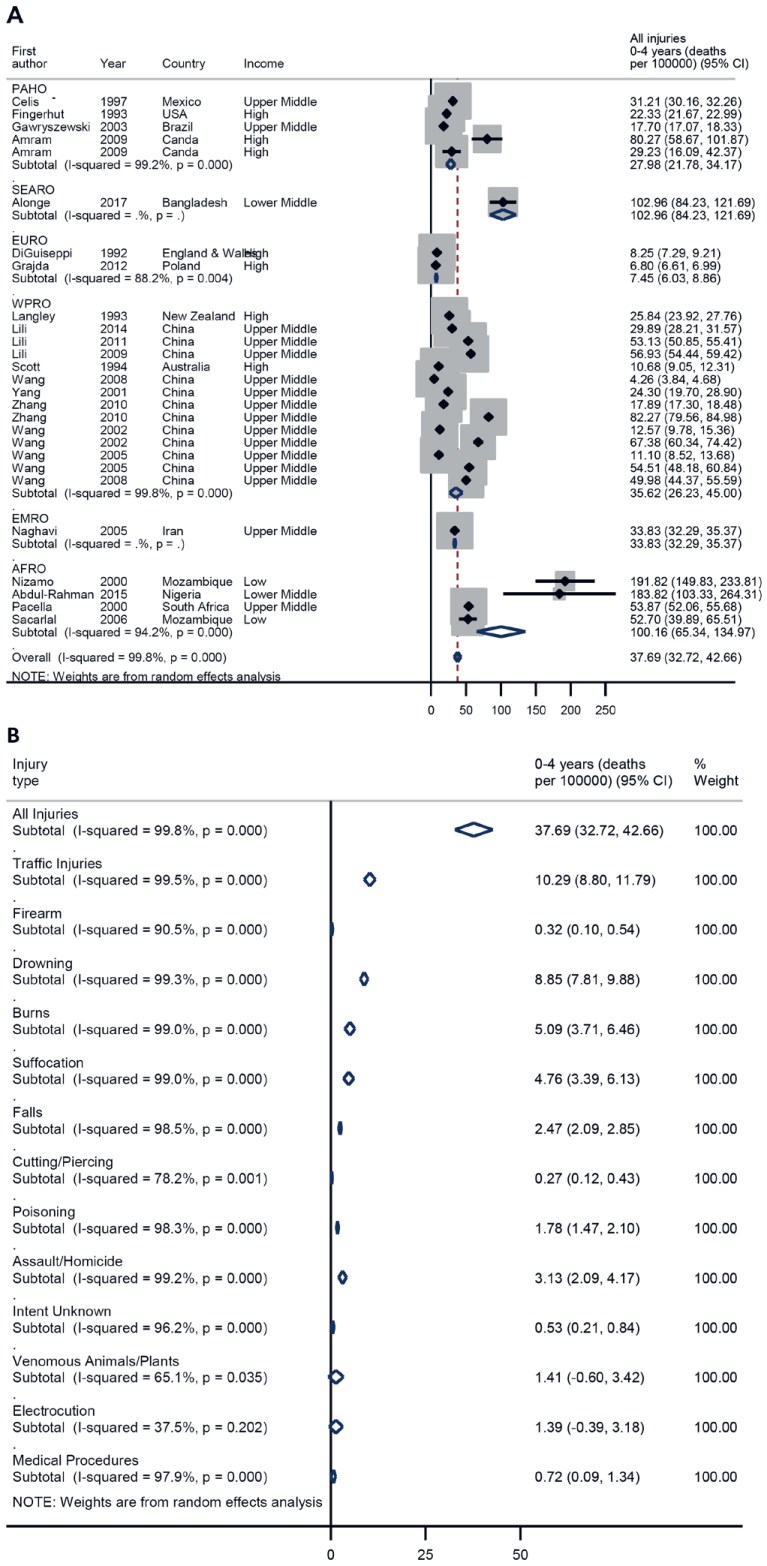
**A.** Pooled crude child injury mortality rate by WHO region, 0-4 years. **B.** Pooled crude child injury mortality rate by type of injury, 0-4 years.

Among children under five years, the pooled injury mortality rate was 37.7 (95% CI = 32.7-42.7) per 100 000 (**Figure 5**, plate A). Traffic injuries and drowning were also the leading causes of deaths in this age-group at 10.3 (95% CI = 8.8-11.8) and 8.9 (95% CI = 7.8-9.9) per 100 000 respectively. Others include burns 5.1 (3.7-6.5) per 100 000, suffocation 4.8 (95% CI = 3.4-6.1) per 100 000, assault 3.1 (95% CI = 2.1-2.9) per 100 000, falls 2.5 (95% CI = 2.1-2.9) per 100 000, poisoning 1.8 (95% CI = 1.5-2.1) per 100 000, venomous animals and plants 1.4 (95% CI = 0.6-3.4) per 100 000 and electrocution 1.4 (95% CI = 0.4-3.2) per 100 000 (**Figure 5**, plate B). Further details on the distribution of crude child injury mortality rates across each WHO region are available from Figures S1-S6 in **Online Supplementary Document(Online Supplementary Document).**

### Estimated global and regional deaths from child injuries

The between study variance (adjusted R^2^) from our model was 30.3%, *P* = 0.037 (**Table 4**). Having accounted for crude child injury mortality changes over age, WHO regions, and study period in our model, we estimated that in 2015 there were 522 167 (95% CI = 395 823-648 630) deaths among children aged 0-4 years, accounting for an adjusted injury mortality rate of 80.5 (95% CI = 61.0-100.0) per 100 000. SEARO recorded the highest number of child injury deaths at 195 084 (95% CI = 159 476-230 502) with an adjusted mortality rate at 105.1 (95% CI = 85.9-124.2) per 100 000. This was closely followed by AFRO with 176 523 (95% CI = 115 040-237 831) deaths, with the adjusted mortality rate being the highest from all regions at 118.0 (95% CI = 76.9-159.0) per 100 000. EURO had the lowest number of under-five injury deaths in 2015 at 13 173 (95% CI = 10 539-15 632), with an adjusted injury mortality rate of 37.6 (95% CI = 22.1-32.8) per 100 000 (**Table 5**).

**Table 4:** Meta-regression model statistics

Under 5 injury mortality (per 100 000)	Coefficient	Standard error	t	*P* > |t|	Lower CI	Upper CI
**reg1 (PAHO)**	-62.72354	23.99989	-2.61	0.017	-112.7864	-12.66065
**reg2 (SEARO)**	-12.92205	41.31005	-0.31	0.758	-99.09331	73.24921
**reg3 (EURO)**	-90.3925	29.76	-3.04	0.007	-152.4708	-28.31423
**reg4 (WPRO)**	-65.96893	20.79313	-3.17	0.005	-109.3426	-22.59521
**reg5 (EMRO)**	-67.67915	37.58827	-1.80	0.087	-146.0869	10.72861
**Year**	1.197741	1.043791	1.15	0.265	-.9795689	3.375052
**_cons (AFRO)**	-2299.962	2091.543	-1.10	0.005	-6662.844	-2062.92
**REML estimate of between-study variance (tau^2^) = 928.7**						
**% residual variation due to heterogeneity (I^2^) = 99.77%**						
**Proportion of between-study variance explained (adjusted R^2^) = 30.33%**						
**Joint test for all covariates, Model F(6,20) = 2.84**						
**With Knapp-Hartung modification = 0.0365**						

AFRO – African region, EMRO – Eastern Mediterranean region, EURO – European region, PAHO – Pan-American Health Organization or American region, SEARO – South East Asia region, WPRO – Western Pacific region, Reg – dummy variables representing each WHO region, t – model probability, cons – model constant (equivalent to AFRO region from model), REML – residual maximum likelihood

**Table 5:** Global and regional child deaths (0-4 y) by injury type in 2015*

Injury type	World		AFRO		EMRO		EURO		PAHO		SEARO		WPRO	
***Rate***	***Deaths***	***Rate***	***Deaths***	***Rate***	***Deaths***	***Rate***	***Deaths***	***Rate***	***Deaths***	***Rate***	***Deaths***	***Rate***	***Deaths***
**Traffic injuries**	21.99	142661	26.14	39110	20.24	13389	7.73	3689	12.44	9279	17.04	31630	10.17	12715
**Drowning**	19.00	123270	6.59	9866	6.25	4135	4.42	2108	7.31	5449	35.31	65533	18.09	22605
**Burns**	10.89	70638	38.63	57784	6.85	4529	5.15	2459	10.67	7953	3.47	6440	1.98	2472
**Suffocation**	10.25	66483	4.48	6695	5.95	3938	3.68	1756	12.44	9279	2.76	5114	7.06	8830
**Falls**	5.34	34626	4.59	6871	4.17	2757	1.84	878	1.38	1031	14.69	27274	5.51	6888
**Poisoning**	3.84	24931	1.18	1762	3.13	2067	1.47	703	1.78	1326	1.84	3409	3.81	4768
**Assault/Homicide**	6.62	42937	15.19	22726	2.23	1477	2.21	1054	6.52	4860	4.49	8334	2.26	2826
**Other injuries†**	2.56	16621	21.20	31711	1.49	985	1.10	527	2.76	2062	25.51	47350	1.41	1766
**All injuries**	**80.50**	**522167**	**118.00**	**176523**	**50.30**	**33276**	**27.60**	**13173**	**55.30**	**41239**	**105.10**	**195084**	**50.30**	**62871**
***Lower CI***	*61.02*	*395823*	*76.90*	*115040*	*48.96*	*32390*	*22.08*	*10539*	*43.06*	*32107*	*85.92*	*159476*	*37.02*	*46270*
***Upper CI***	*99.99*	*648630*	*158.98*	*237831*	*52.64*	*34822*	*32.75*	*15632*	*67.55*	*50370*	*124.18*	*230502*	*63.58*	*79472*

AFRO – African region, EMRO – Eastern Mediterranean region, EURO – European region, PAHO - Pan-American Health Organization or American region, SEARO – South East Asia region, WPRO – Western Pacific region

*Estimates based on meta-regression model.

†Includes asphyxiation, firearms, cutting/piercing, venomous animal/plants, electrocution and medical procedures.

Globally, traffic injuries and drowning were the leading causes of under-five injury fatalities in 2015 with 142 661 (22.0 per 100 000) and 123 270 (19.0 per 100 000) child deaths, respectively. Burns were the leading causes of child injury fatalities in AFRO (57784 deaths), drowning in WPRO and SEARO (22 605 and 65 533 deaths, respectively), and suffocation jointly leading with traffic injuries in PAHO (9279 deaths).

## DISCUSSION

This study provided the first estimates of the mortality due to injuries, and by specific cause, among children under five years across WHO regions. Several authors have noted that child survival has increased globally, but with uneven progress persisting across many developing countries [2,5]. There are still doubts on the trend in fatalities from child injuries over the years, and how they compare to the decrease observed from other important causes of child mortality [4,8]. This is due to predominantly limited data and research on child injuries across many world settings [13,49]. Our findings thus, among others, highlight the need for renewed efforts from all stakeholders towards addressing a rather hidden burden of child injuries globally.‬‬‬‬‬‬‬‬‬‬‬‬‬‬‬‬‬‬‬‬‬‬‬‬‬‬‬‬‬‬‬‬‬‬‬‬‬‬‬‬‬‬‬‬‬‬‬‬‬‬‬‬‬‬‬‬‬‬‬‬‬‬‬‬‬‬‬‬‬‬‬‬‬‬‬‬‬‬‬‬

Our global estimate of over 522 000 (80.5/100 000) children under five years who died due to injuries in 2015 is higher than previously reported. Liu and colleagues [5] estimated over 327000 deaths in 2015, with an upper limit of about 410 000. Our model was primarily based on WHO regions, and with the individual regional estimates contributing to the global estimates. We believe the relatively higher global estimate provided in this study are particularly driven by the higher number of deaths in Africa and South-East Asia. The estimates in these two regions are plausible as previous studies have reported that the two regions contribute highest to overall global child mortality [2]. Besides, the estimate given by Liu and colleagues did not include deaths due to injuries in the neonatal period. We therefore could expect that as a quarter of world’s livebirths was recorded in sub-Saharan Africa in 2015, accidental deaths (*eg* from asphyxia, at 189/100 000 in this study) in the neonatal period would be relatively higher in this region, ultimately contributing to the high number of deaths we reported. One other factor for the relatively higher estimate is the fact that we reported a greater number of child injury deaths by specific cause (seven) than reported previously, with a small fraction of child deaths (at 2.56/100 000), labelled as “others”, that could not be accounted for (**Table 5**). For example, the WHO reported an injury mortality of 38.8/100 000 among children under 20 years in 2008 [1], and the category labelled “others” was as high as 34.3% of all deaths at 13.3/100 000 [1]. This implies that with more leading specific causes of child injury deaths reported separately in this study, our overall global estimate for all injuries could expectedly be higher.

Our estimates of higher child deaths from traffic injuries and drowning, representing 27.3% and 23.6% of all injury deaths, have been well documented in previous studies [1,2]. In 2008, the WHO reported that traffic injuries and drowning were the leading causes of deaths among children globally, with an estimated mortality rate of 10.7 and 7.2 per 100 000, respectively among children aged 0-18 years. Drowning was particularly a leading cause of death in the WPRO and SEARO with 22 650 and 65 533 deaths, respectively. Chan et al [17] noted that drowning was the leading cause of injury deaths in China, with about 11 000 deaths in children under five years in 2010, which if expressed in terms of the entire WPRO and demographic changes since then, may be relatively similar to our estimate. According to Chandran et al. [3] drowning is an important cause of unintentional injury deaths accounting for 19% of all injury deaths in children under five years, which is relatively in the range of our proportion of 23.6%.While prevailing unsafe roads with high rates of pedestrian accidents in many LMICs may be attributable to the high deaths from traffic injuries among children [16], the increased access to unfenced water sources in rural areas, and lack of close supervision and unavailability of life jackets at swimming pools and related recreational sites are leading risks for high number of child deaths from drowning in many settings [2]. Addressing safety on world roads and at recreational sites are important steps towards reducing child deaths from injuries.

Meanwhile, in the African region, burns were the leading causes of child injury deaths accounting for 33% of all deaths, compared to traffic injuries at 22%. This is an important finding in this study which underscores a need to address exposures to fire and flames more keenly. Across many LMICs, children aged 1-4 years have been disproportionately affected by burns [50], as children are at increased risk due to prevalent outdoor cooking and use of firewood in rural settings, asides other challenges from limited parental supervision and literacy [51]. The WHO reported that rate of deaths from fire and flames among children in LMICs is close to 11 times higher than recorded in HICs [1].

Although we have attempted to provide improved estimates of child deaths by different causes of injuries and across WHO regions, it is worthwhile to note that our estimates are limited by widespread disparities in child deaths, a challenge that is well situated in many reports. For example, in 2008, Black et al [52] estimated that injuries accounted for 279 000 deaths among children aged 1-4 years, with an upper limit of 738000. In the same year, the WHO estimated 950 000 deaths from injuries among children aged 0–18 years, with mortality among 1-4 years at 45.8/100 000 [1]. In 2015, the GBD collaborators reported even lower estimates of child deaths from injuries with about 180 000 deaths estimated in children aged 0-4 years [2]. These disparities are occasioned by varying study designs, case definitions and limited data from many LMICs, which clearly reflected in the distribution of the pooled crude rates for the different age groups, and the high heterogeneity when our data were aggregated. Besides, with several countries in the South-East Asia and African region not represented, the generalization of these estimates may be somewhat limited.

## CONCLUSION

Our findings suggest deaths from child injuries are higher than previously reported. Knowledge and awareness are crucial steps in the response to child deaths from injuries, as several interventions are currently skewed towards infectious, neonatal and nutritional causes. Countries need to leverage on existing low-cost information systems and the established national demographic surveillance to collate and process data on child injuries to inform the needed policy reforms. Moreover, educational campaigns, good road designs and legislation, swimming lessons with necessary safety precautions, and availability of safer homes, schools and communities are proven strategies. We believe the findings of this study are revealing and may have provided better understanding on child injury death profiles needed to guide further research, policy reforms and relevant strategies to reduce this burden across world regions.
