# Supplementary material for: Global and regional child deaths due to injuries: an assessment of the evidence
Source: J Glob Health. 2018 Nov 29;8(2):021104. doi: 10.7189/jogh.08.021104 (PMC6317703; doi:10.7189/jogh.08.021104)

Online Supplementary Document

Adeloye et al. Explaining progress towards Millennium Development Goal 4 for child survival in Tanzania.

J Glob Health 2018;8:021104

Table S1. Search terms

1. incidence.mp. or Incidence/
2. Child Mortality/ or Infant Mortality/ or mortality.mp. or Mortality/
3. fatal outcome.mp. or Fatal Outcome/
4. questionnaires.mp. or Questionnaires/
5. population surveillance.mp. or Population Surveillance/
6. fatality.mp.
7. Death/ or "Cause of Death"/ or death.mp.
8. 1 or 2 or 3 or 4 or 5 or 6 or 7
9. injur*.mp.
10. Accident Prevention/ or accident*.mp.
11. Accidents, Traffic/ or Accidents/ or Accidents, Home/
12. Accidental Falls/ or "Wounds and Injuries"/
13. Near Drowning/ or Drowning/
14. Snake Bites/ or "Bites and Stings"/
15. Burns, Chemical/ or Burns/ or Burns, Inhalation/ or Burns, Electric/
16. Poisoning/
17. fall*.mp.
18. burn*.mp.
19. poison*.mp.
20. drown*.mp.
21. traffic accident.mp.
22. bite*.mp.
23. sting*.mp.
24. 9 or 10 or 11 or 12 or 13 or 14 or 15 or 16 or 17 or 18 or 19 or 20 or 21 or 22 or 23
25. Developing Countries/
26. low income country.mp.
27. low income countries.mp.
28. middle income country.mp.
29. middle income countries.mp.
30. africa/ or africa, northern/ or algeria/ or egypt/ or libya/ or morocco/ or tunisia/ or "africa south of the sahara"/ or africa, central/ or cameroon/ or central african republic/ or chad/ or congo/ or "democratic republic of the congo"/ or gabon/ or africa, eastern/ or burundi/ or djibouti/ or eritrea/ or ethiopia/ or kenya/ or rwanda/ or somalia/ or sudan/ or tanzania/ or uganda/ or africa, southern/ or angola/ or botswana/ or lesotho/ or malawi/ or mozambique/ or namibia/ or south africa/ or swaziland/ or zambia/ or zimbabwe/ or africa, western/ or benin/ or burkina faso/ or cape verde/ or gambia/ or ghana/ or guinea/ or guinea-bissau/ or liberia/ or mali/ or mauritania/ or niger/ or nigeria/ or senegal/ or sierra leone/ or togo/ or "antigua and barbuda"/ or cuba/ or dominica/ or dominican republic/ or grenada/ or haiti/ or jamaica/ or saint lucia/ or "saint vincent and the grenadines"/ or central america/ or belize/ or costa rica/ or el salvador/ or guatemala/ or honduras/ or nicaragua/ or panama/ or panama canal zone/ or mexico/ or south america/ or argentina/ or bolivia/ or brazil/ or chile/ or colombia/ or ecuador/ or guyana/ or paraguay/ or peru/ or suriname/ or uruguay/ or venezuela/ or asia/ or asia, central/ or

kazakhstan/ or kyrgyzstan/ or tajikistan/ or turkmenistan/ or uzbekistan/ or cambodia/ or east timor/ or indonesia/ or laos/ or malaysia/ or myanmar/ or philippines/ or thailand/ or vietnam/ or asia, western/ or bangladesh/ or bhutan/ or india/ or sikkim/ or middle east/ or afghanistan/ or iran/ or iraq/ or jordan/ or lebanon/ or syria/ or turkey/ or yemen/ or nepal/ or pakistan/ or sri lanka/ or exp china/ or korea/ or "democratic people's republic of korea"/ or mongolia/ or albania/ or latvia/ or lithuania/ or bosnia-herzegovina/ or bulgaria/ or "macedonia (republic)"/ or moldova/ or montenegro/ or "republic of belarus"/ or romania/ or exp russia/ or serbia/ or ukraine/ or transcaucasia/ or armenia/ or azerbaijan/ or "georgia (republic)"/ or comoros/ or madagascar/ or mauritius/ or seychelles/ or melanesia/ or fiji/ or papua new guinea/ or vanuatu/ or micronesia/ or palau/ or samoa/ or american samoa/ or tonga/
31. kiribati.mp.
32. kosovo.mp.
33. marshall islands.mp.
34. solomon islands.mp.
35. south sudan.mp.
36. maldives.mp.
37. tuvalu.mp.
38. "west bank and gaza".mp.
39. "sao tome and principe".mp.
40. 25 or 26 or 27 or 28 or 29 or 30 or 31 or 32 or 33 or 34 or 35 or 36 or 37 or 38 or 39
41 8 and 25 and 40

Table S2: Quality assessment

First author	Study design	Sampling	Statistical analysis	Limitations	Overall grade	Assessment
**Nizamo**	1	1	0	0	2	Moderate
**Abdul-Rahman**	1	1	0	0	2	Moderate
**Pacella**	1	1	1	0	3	Moderate
**Sacarlal**	1	1	1	0	3	Moderate
**Naghavi**	1	1	1	0	3	Moderate
**DiGuiseppi**	1	1	1	1	4	High
**Grajda**	1	1	1	1	4	High
**Celis**	1	2	1	1	5	High
**D'Agostini**	1	1	1	1	4	High
**Espitia-Hardeman**	1	2	1	1	5	High
**Fingerhut**	1	2	1	1	5	High
**Gawryszewski**	1	2	1	1	5	High
**Aldana**	1	1	1	1	4	High
**Amram**	1	2	1	1	5	High
**Clemens**	1	2	1	1	5	High
**Rahman**	1	1	1	1	4	High
**Jagnoor**	1	2	1	1	5	High
**Alonge**	1	2	1	1	5	High
**Huo**	1	1	1	1	4	High
**Langley**	1	2	1	1	5	High
**Lili**	1	2	1	1	5	High
**Scott**	1	2	1	1	5	High
**Wallis**	1	2	1	1	5	High
**Wang**	1	1	1	0	3	Moderate
**Yang**	1	1	1	0	3	Moderate
**Zhang**	1	1	1	0	3	Moderate
**Zhang**	1	1	1	0	3	Moderate
**Wang**	1	1	1	0	3	Moderate
**Hayman**	0	1	1	1	3	Moderate

Table S3: Papers excluded during the quality criteria stage

AKBARI, M.E., NAGHAVI, M., & SOORI, H. (2006) Epidemiology of deaths from injuries in the Islamic Republic of Iran. *La Revue de Santé de la Méditerranée orientale*. 12 (3-4), 382-390.
ANTUNES, J.L. & WALDMAN, E.A. (2002) Trends and spatial distribution of deaths of children aged 12-60 months in São Paulo, Brazil, 1980-1998. *Bulletin of the World Health Organization*. 80(5), 391-340.
ARMOUR-MARSHALL, J., WOLFE, I., RICHARDSON, E., KARANIKOLOS, M., & MCKEE, M. (2012) Childhood deaths from injuries: trends and inequalities in Europe. *European Journal of Public Health*. 22(1), 61-65.
BLANK, D. & LIBERAL, E.F. (2005) Pediatricians and external causes of morbidity and mortality. *Journal de Pediatria.* 81(5 suppl), S119-S122.
BURROWS, S., VAN NIERKERK, A., & LAFLAMME, L. (2010) Fatal injuries among urban children in South Africa: risk distribution and potential for reduction. *Bulletin of the World Health Organization*. 88, 267-272 [online]. Available at <http://www.who.int/bulletin/volumes/88/4/09-068486.pdf /> [Accessed 16th August 2013].
CARDONA, M., JOSHI, R, IVERS, R.Q., IYENGAR, S., CHOW, C.K., COLMAN, S., RAMAKRISHNA, G., DANDONA, R., STEVENSON, M.R., & NEAL, B.C. (2008) The burden of fatal and non-fatal injury in rural India. *Injury Prevention*. 14, 232-237.
CHOWDHURY, S.M., RAHMAN, A., MASHREKY, S.R., GIASHUDDIN, S.M., SVANSTRÖM, L., HÖRTE, L.G., & RAHMAN, F. (2009) The Horizon of Unintentional Injuries among Children in Low-Income Setting: An Overview from Bangladesh Health and Injury Survey. *Journal of Environmental and Public Health*. 1-6.
DHILLON, S., MAHAJAN, A., & SEKHON, H.S. (2012) Fatal Childhood Injuries in Shimla Hills. *Medico-Legal Update*. 12(2), 155-157.
DONALDSON, R.I., HUNG, Y.W., SHANOVICH, P, HASOON, T., & EVANS, G. (2010) Injury burden during an insurgency; the untold trauma of infrastructure breakdown in Baghdad, Iraq. *The Journal of Trauma.* 69(6), 1379-1385.
DONROE, J., GILMAN, R.H., BRUGGE, D., MWAMBURI, M., & MOORE, D.A.J. (2009) Falls, poisonings, burns, and road traffic injuries in urban Peruvian children and adolescents: a community based study. *Injury Prevention*. 15, 390-396
GARRIB, A., HERBST, A.J., HOSEGOOD, V., & NEWELL, M.L. (2011) Injury mortality in rural South Africa 2000-2007: rates and associated factors*. *Tropical Medicine and International Health*. 16(4), 439-446.
GAWRYSZEWSKI, V.P. (2007) Injury mortality report for São Paulo State, 2003. *São Paulo Medical Journal*. 125(3), 139-143.
HALDER, A. K., GURLEY, E.S., NAHEED, A., SAHA, S.K., BROOKS, W.A., ARIFEEN, S.E., SAZZAD, H.M.S., KENAH, E., & LUBY, S.P. (2009) Causes of Early Childhood Deaths in Urban Dhaka, Bangladesh. *PLoS ONE*. 4(12) [online]. Available at < http://www.plosone.org/article/fetchObject.action?uri=info%3Adoi%2F10.1371%2Fjournal.pone.0008145&representation=PDF> [Accessed 15th August 2013].
HU, G., BAKER, S.P., & BAKER, T.D. (2010) Urban- Rural Disparities in Injury Mortality in China, 2006. *The Journal of Rural Health*. 26, 73-77.
HUONG, N. T. L., TU, N. T.H., MORITA, S., & SAKAMOTO, J. (2008) Injury and pre-hospital trauma care in Hanoi, Vietnam. *Injury International Journal of the Care of the Injured*. 39, 1026-1033.
JETSRISUPARB, A., TEERATAKULPISARN, J., WERAARCHAKUL, W., THEPSUTHAMMARAT, K., & SUTRA, S. (2012) Health situation analysis of Thai Children aged 1-5 years in 2010: implications for health education and health service reform. *Journal of the Medical Association of Thailand*. 95(Supplement 7), S30-S42.
KALAISELVAN, G., DONGRE, A.R., & MAHALAKSHMY, T. (2011) Epidemiology of injury in rural Pondicerry, India. *Journal of Injury and Violence Research*. 3(2), 62-67.
KOBUSINGYE, O., GUWATUDDE, D., & LETT, R. (2001) Injury patterns in rural and urban Uganda. *Injury Prevention* 7, 46-50.
LASI, S., RAFIQUE, G., & PEERMOHAMED, H. (2010) Childhood injuries in Pakistan: Results from Two Communities. *Journal of Health, Population and Nutrition*. 28(4): 392–398.
LINNAN, M., PHAM, C.V., LE, L.C., LE, P.N. & LE, A.V. (eds) (2003) *Report to UNICEF on the Vietnam Multi-*

center Injury Survey. Hanoi School of Public Health, Vietnam [online]. Available at < http://www.tasc-gcipf.org/downloads/Vietnam%20-%20UNICEFfinalVMISreportfinal.pdf> [Accessed 15th August 2013].

LINNAN, M., REINTEN, T., & WEI, J.R. (2009*) A final report to UNICEF Cambodia on The Cambodia Accident and Injury Survey, 2007*. The Alliance for Safe Children, Bangkok, Thailand [online]. Available at < Part 1: http://www.unicef.org/cambodia/Cambodia_Accident_and_Injury_Survey_Technical_Child_Injury_Report_2007_Part1.pdf Part 2: http://www.unicef.org/cambodia/Cambodia_Accident_and_Injury_Survey_Technical_Child_Injury_Report_2007_Part2.pdf> [Accessed 15th August 2013].

LIU, Q., ZHANG, L., LI, J., ZUO, D., KONG, D., SHEN, X., GUO, Y., & ZHANG, Q. (2012) The gap in injury mortality rates between urban and rural residents of Hubei province, China. *BMC Public Health*. 12(180) [online]. Available at < http://www.biomedcentral.com/content/pdf/1471-2458-12-180.pdf> [Accessed 15th August 2013].

MAMADY, K., YAO, H., ZHANG, X., XIANG, H., TAN, H., & HU, G. (2012) The injury mortality burden in Guinea. *BMC Public Health*. 12(733) [online]. Available at < http://www.biomedcentral.com/content/pdf/1471-2458-12-733.pdf> [Accessed 15th August 2013].

NAVARATNE, K.V., FONSEKA, P., RAJAPAKSHE, L., SOMATUNGA, L., AMERATUNGA, S., IVERS, R., & DANDONA, R. (2009) Population-based estimates of injuries in Sri Lanka. *Injury Prevention*. 15, 170-175.

NORMAN, R., MATZOPOULOS, R., GROENEWALD, P., & BRADSHAW, D. (2007) The high burden of injuries in South Africa. *Bulletin of the World Health Organization* 85(9), 695-702 [online]. Available at < http://www.ncbi.nlm.nih.gov/pmc/articles/PMC2636399/pdf/06-037184.pdf [Accessed 16th August 2013].

OLAWALE, O. A., & OWOAJE, E.T. (2007) Incidence and pattern of injuries among residents of a rural area in South-Western Nigeria: a community-based study. *BMC Public Health*. 7(246) [online]. Available at < http://www.ncbi.nlm.nih.gov/pmc/articles/PMC2169237/pdf/1471-2458-7-246.pdf> [Accessed 15th August 2013]

POOROLAJAL, J., CHERAGHI, P.C., HAZAVEHEI, S.M.M., & REZAPUR-SHANHKOLAI, F. (2013) Factors Associated with Mothers' Beliefs and Practices Concerning Injury Prevention in Under Five-Year Children, Based on Health Belief Model. *Journal of Research in Health Sciences*. 13(1), 63-68.

RATTANAMONGKOLGUL, S., & PLITPONKARNPIM, A. (2012) Incidence and patterns of registered injuries among children in Ongkharak District, Nakhon Nayok, Thailand. *Journal of the Medical Association of Thailand*. 95(Supplement 12), S118-S124.

SENGOELGE, M., HASSELBERG, M., & LAFLAMME, L. (2010) Child home injury mortality in Europe: a 16 country analysis. *European Journal of Public Health*. 21(2), 166-170.

SITTHI-AMORN, C., CHAIPAYOM, O., UDOMPRASERTGUL, V., LINNAN, M., DUNN, T., BECK, L., CARDENAS, V., IRVINE, K., FORSGATE, D., & COX, R. (2006) *Child injury in Thailand: A report on the Thai National Injury Survey. Bangkok, Thailand* [online]. Available at < http://www.tasc-gcipf.org/downloads/Thai%20child%20report.pdf> [Accessed 15th August 2013].

STEWART, K.A.A., GROEN, R.S., KAMARA, T.B., FARAHZAD, M.M., SAMAI, M., CASSIDY, L.D., KUSHNER, A.L., & WREN, S.M. (2013) Traumatic Injuries in Developing Countries Report from a Nationwide Cross-sectional survey of Sierra Leone. *JAMA Surgery*. 148(5), 463-9.

STRUKCINSKIENE, B. (2008) Unintentional injury mortality trends in children and adolescents in Lithuania between 1971 and 2005. *International Journal of Injury Control and Safety Promotion*. 15(1), 1-8.

SWART, L.A., & SEEDAT, M (2001) An epidemiological study of injury in a low socioeconomic context: implications for prevention. *Injury Control and Safety Promotion*. 8(4), 241-249.

TERCERO, F. ANDERSSON, R., PEÑA, R., ROCHA, J., & CASTRO, N. (2006) The epidemiology of moderate and severe injuries in a Nicaraguan community: A household-based survey. *Public Health*. 120, 106-114.

THE UNITED NATIONS CHILDREN'S FUND (UNICEF) (2007*) Innocenti Working Papers Child Mortality and Injury in Asia*. UNICEF Innocenti Research Centre, Italy [online]. Available at < http://www.tasc-gcipf.org/downloads/Innocenti_Papers_2008.pdf> [Accessed 16th August 2013].

TOBLIN, R.L., BRENNER, R.A., TANEJA, G.S., ROSSI, M.W., COLLINS, M., MICKALIDE, A.D., OVERPECK, M.D., CLINTON-REID, Y., DEVVER, J.A., BOYLE, K., TRUMBLE, A.C., SCHEIDT, P.C. (2011) Preventing Yong Children's Injuries: Analysis of Data from a Population-Based Surveillance. *Maternal and Child Health Journal*. 15, S35-S41.

WANG, Y., HE, C., LI, X., MIAO, L., ZHU, J., & LIANG, J. (2012) Nationwide study of injury-related deaths among children aged 1-4 years in China, 2000-2008. *Journal of Peadiatrics and Child Health* [online] via Edinburgh University login at < http://onlinelibrary.wiley.com/doi/10.1111/j.1440-1754.2012.02525.x/full> [Accessed 10th August 2013].

Figure S1. Pooled crude child injury deaths, AFRO.

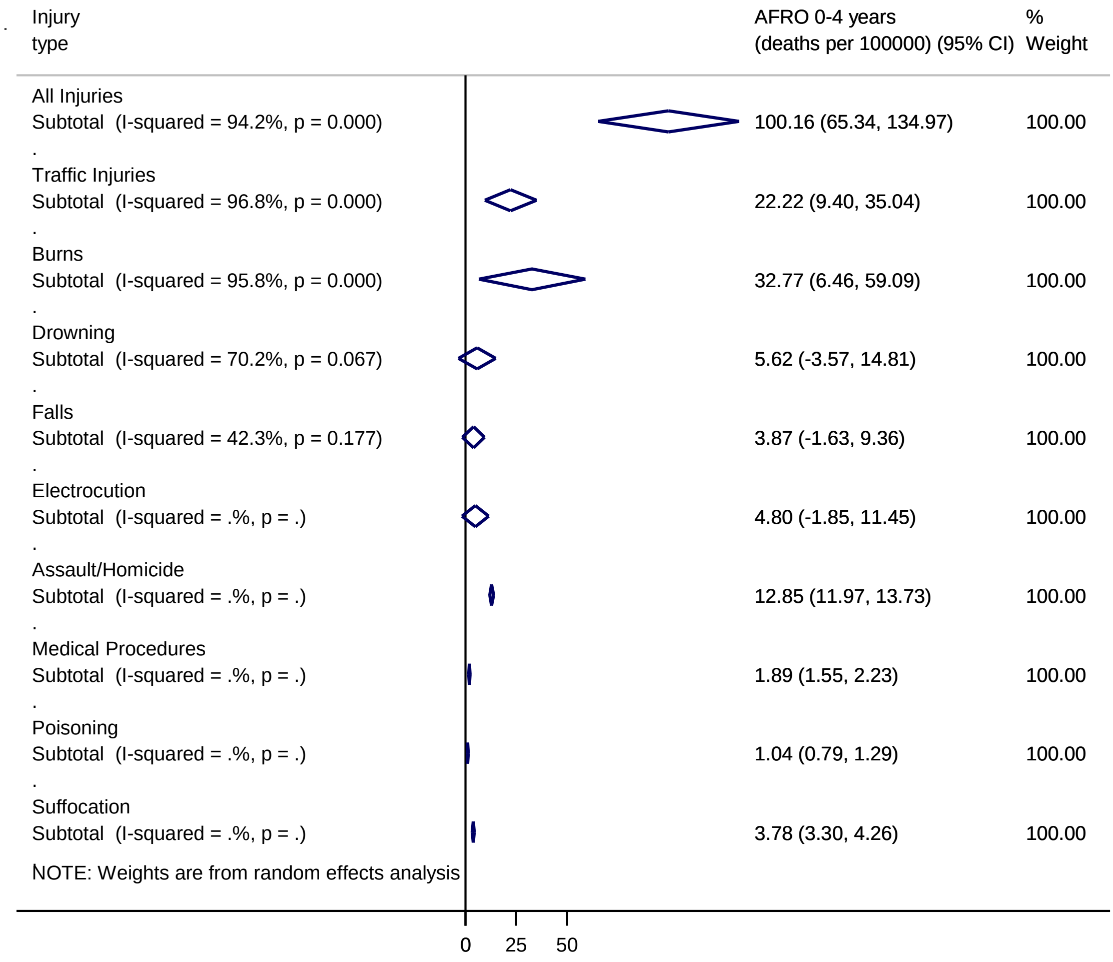

Figure S2. Pooled crude child injury deaths, EMRO.

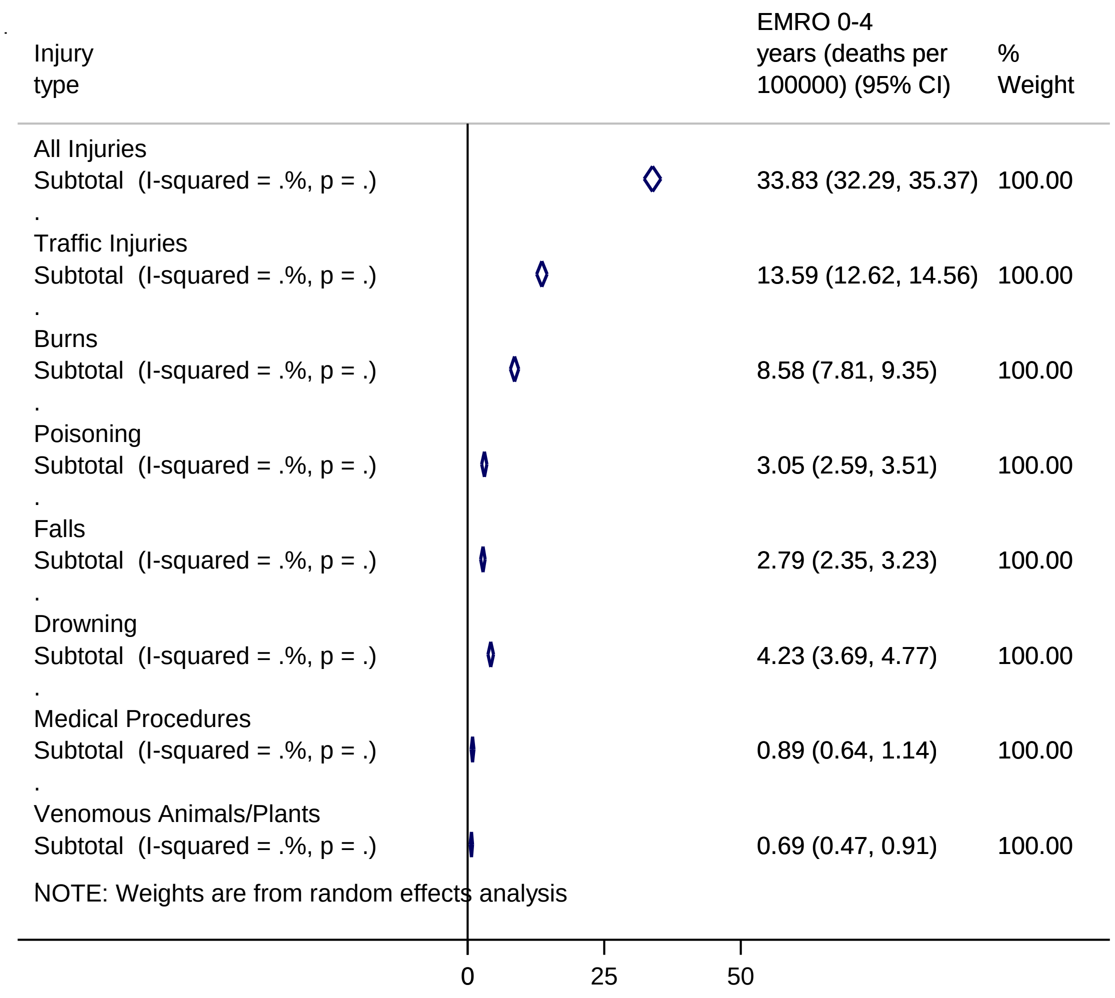

Figure S3. Pooled crude child injury deaths, EURO.

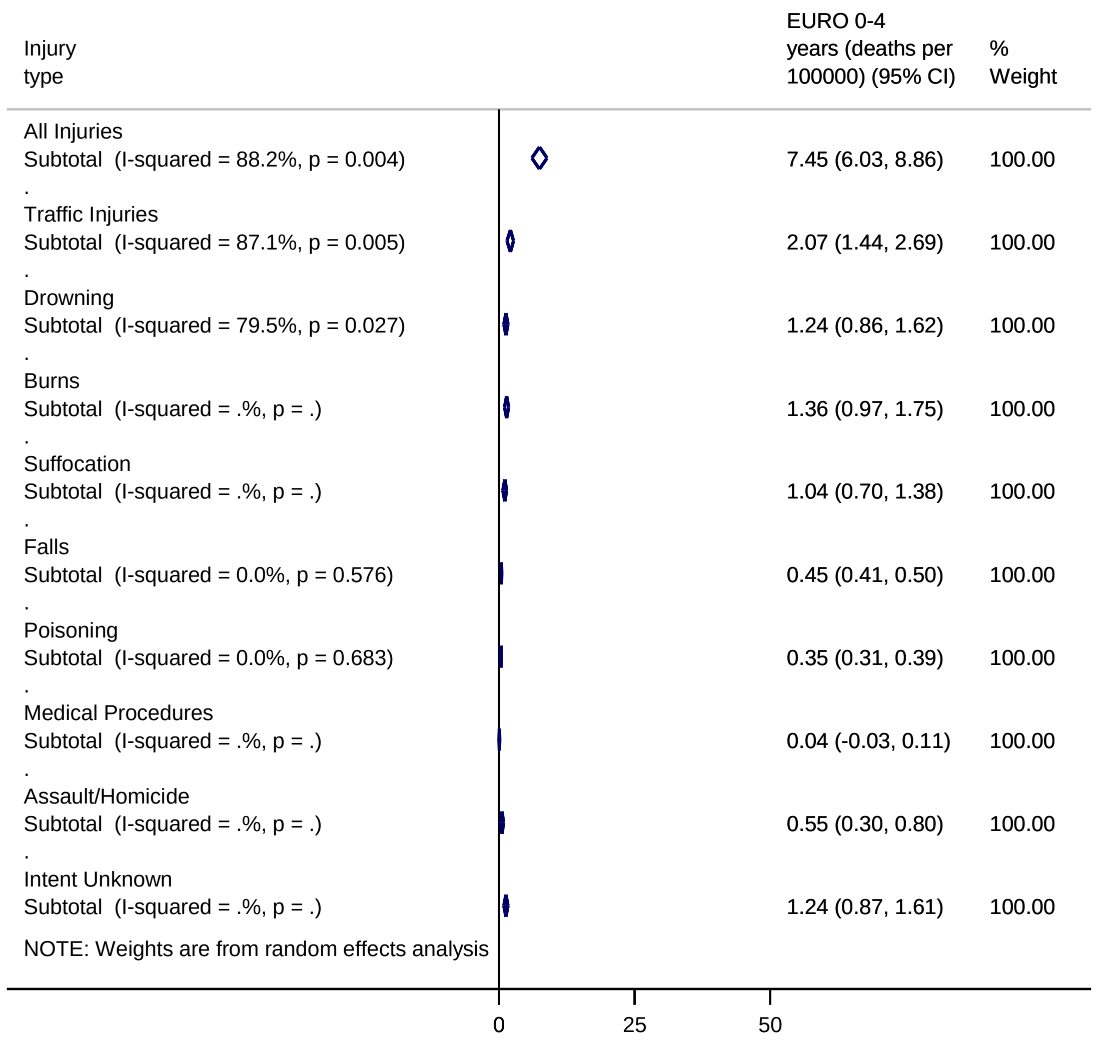

Figure S4. Pooled crude child injury deaths, PAHO.

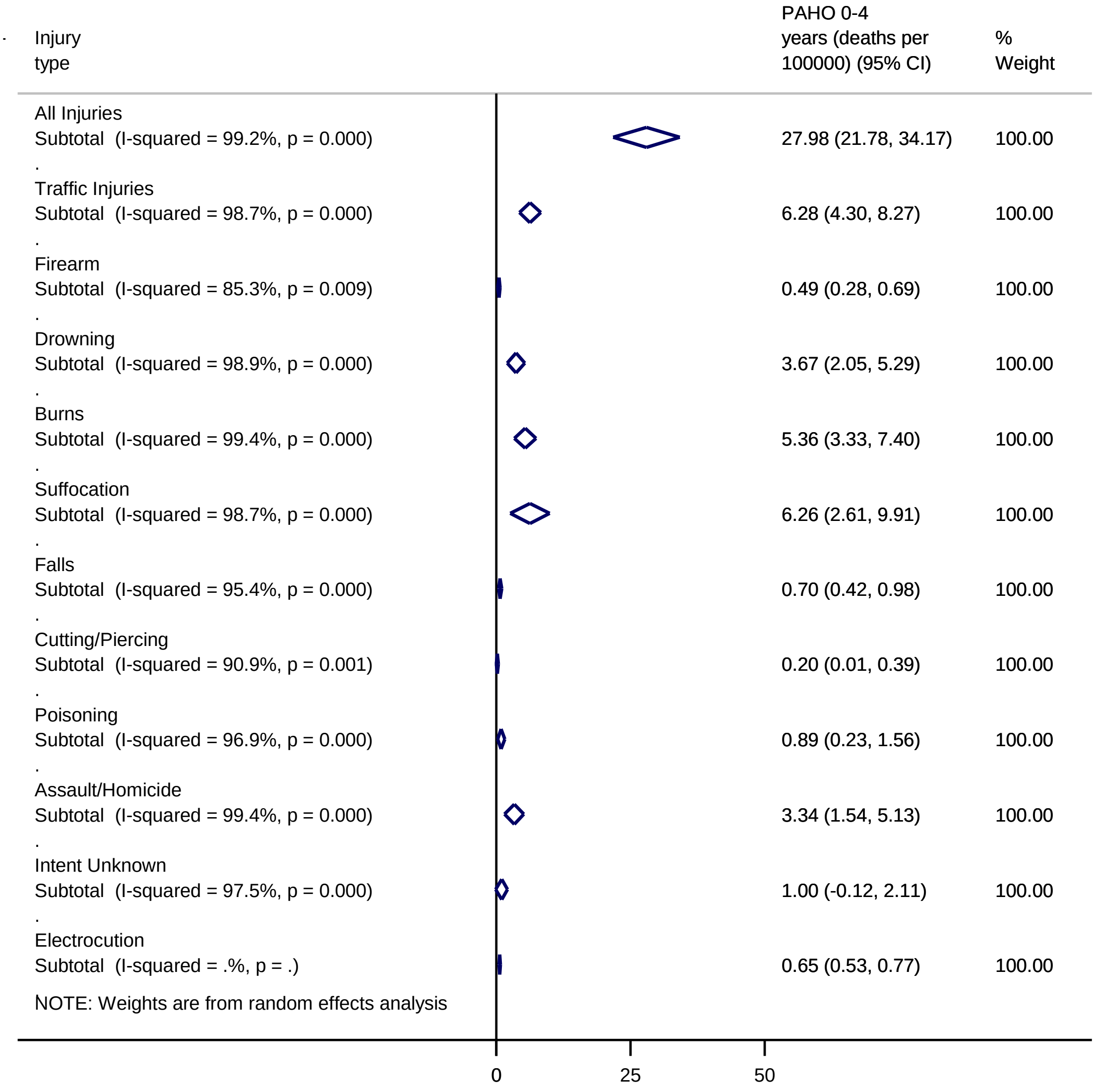

Figure S5. Pooled crude child injury deaths, SEARO.

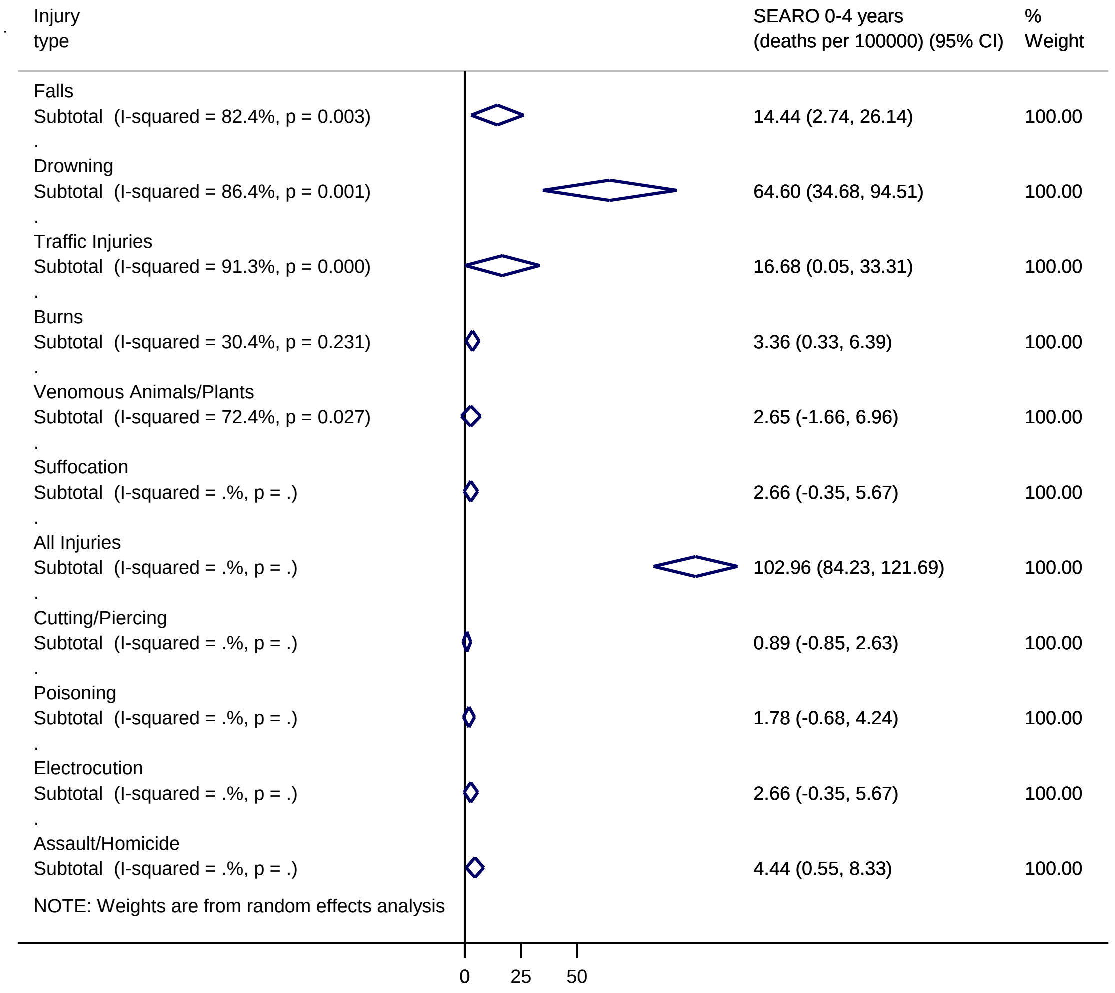

Figure S6. Pooled crude child injury deaths, WPRO.

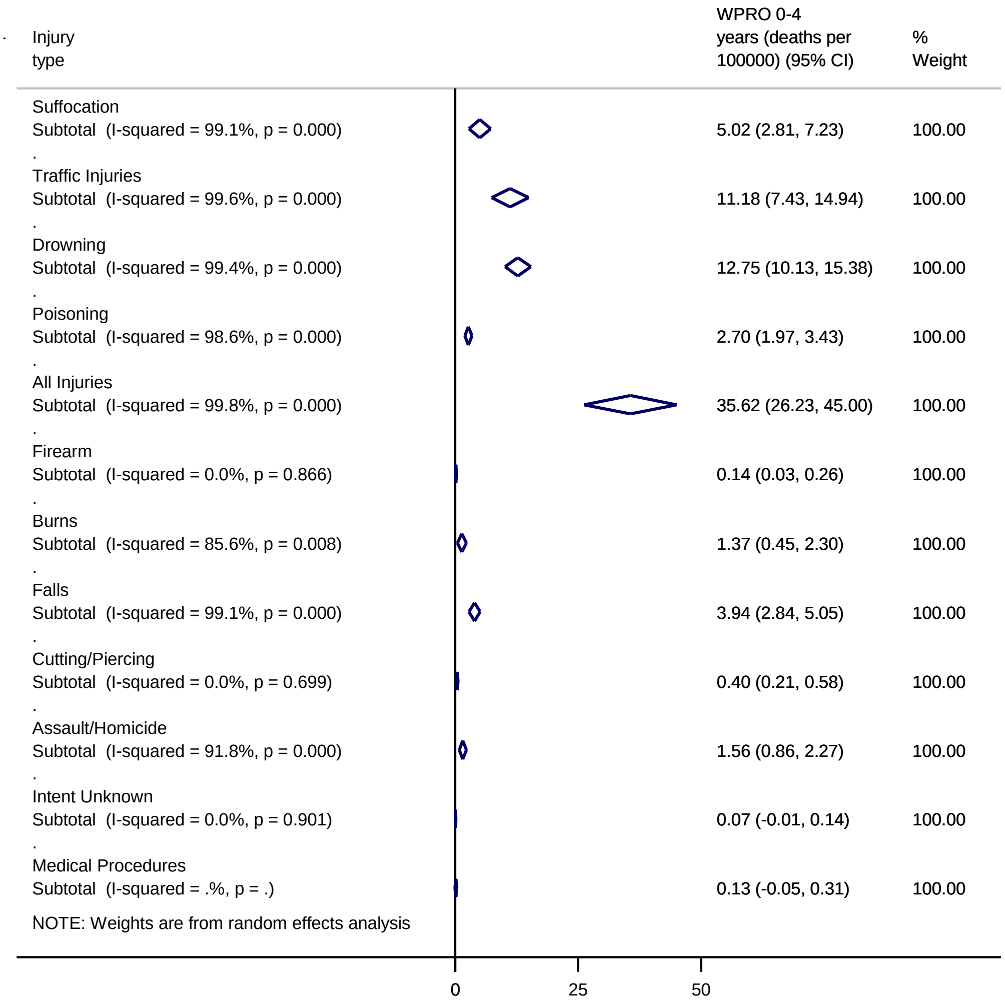

Supplement: Online Supplementary Document [file jogh-08-021104-s001.pdf]
